# Implementing federated learning for privacy-preserving emotion detection in educational environments

**DOI:** 10.3389/frai.2025.1644844

**Published:** 2025-11-07

**Authors:** Rommel Gutiérrez, William Villegas-Ch, Sergio Luján-Mora

**Affiliations:** 1 Escuela de Ingeniería en Ciberseguridad, FICA, Universidad de Las Américas, Quito, Ecuador; 2 Departamento de Lenguajes y Sistemas Informáticos, Universidad de Alicante, Alicante, Spain

**Keywords:** federated learning, emotion detection, data privacy, educational environments, artificial intelligence

## Abstract

Emotion detection has become an essential tool in educational settings, where understanding and responding to students’ emotions is crucial to improving their engagement, academic performance, and emotional well-being. However, traditional emotion detection systems, such as DeepFace, and hybrid transformer-based models face significant data privacy and scalability limitations. These models rely on transferring sensitive data to central servers, compromising student confidentiality and making deployment in large or diverse populations difficult. In this work, we propose a federated learning-based model designed to detect emotions in educational settings, preserving data privacy by processing them locally on students’ devices (smartphones, tablets, and laptops). The model was integrated into the Moodle platform, allowing its evaluation in a conventional educational environment. Advanced anonymization and preprocessing techniques were implemented to ensure the security of emotional data and optimize its quality. The results demonstrate that the proposed model achieves a precision of 87%, a recall of 85%, and an F1-score of 86%, maintaining its performance under adverse conditions, such as low lighting and ambient noise. In addition, a 15% increase in academic participation and a 12% improvement in the average academic performance of students were observed, highlighting the system’s positive impact on educational dynamics. This innovative method combines privacy, scalability, and performance, positioning itself as a viable and sustainable solution for emotion detection in contemporary educational environments.

## 1 Introduction

Emotion detection has emerged as a critical area in developing intelligent systems, particularly in educational contexts, where emotions play a pivotal role in student learning and behavior (Mutawa and Hassouneh, 2024; Shmelova et al., 2024). Understanding and responding to student emotions can significantly improve the personalization of teaching strategies, optimize academic engagement and performance, and contribute to the overall emotional well-being of students (Elisondo et al., 2024). However, the implementation of emotion detection systems faces significant challenges related to data privacy, scalability, and integration into diverse educational settings (Wang A. et al., 2024).

Centralized models, such as DeepFace by An et al. (2023) facial features have been widely used for emotion detection due to their high performance on metrics such as precision and F1-score. However, these systems require the transfer of sensitive data to external servers for training and inference, which poses significant privacy risks. In contrast, models based on Takase and Kiyono (2023) transformers have demonstrated their ability to integrate multiple modalities, such as text, audio, and images, offering a more robust approach. Despite their advantages, high computational costs and configuration complexity limit their implementation in educational settings.

In education, privacy and accessibility are crucial factors. Transferring students’ emotional data to external servers compromises confidentiality and poses ethical and legal challenges in handling sensitive information (Lee et al., 2024). At the same time, accessibility refers to the ability of emotion detection systems to function effectively across diverse educational contexts, including institutions with limited infrastructure or students with varying levels of technological access. Systems that require high-performance computing or stable connectivity may exclude part of the student population, reinforcing educational inequality. Despite these limitations, few studies have addressed these issues by developing emotion detection systems specifically designed to be integrated into learning platforms, such as Moodle, ensuring both data protection and adaptability to the technological realities of educational institutions (Labidi et al., 2021; Woodward et al., 2024).

This work introduces a model based on federated learning designed for emotion detection in educational settings, which addresses these critical challenges. Federated learning allows training models to be run directly on users’ local devices, eliminating the need to transfer sensitive data to central servers (Sengupta et al., 2024). In addition to model development, this work emphasizes the practical integration of the federated emotion detection system into real educational environments, assessing its influence on student engagement and academic outcomes. This feature improves privacy and enables greater scalability by allowing the system to operate on large and heterogeneous student populations (Wang et al., 2024a).

The proposed methodology includes a multi-stage approach, starting with the collection of emotional data through images, audio, and text generated during academic activities. The data was preprocessed using advanced anonymization and feature extraction techniques, such as random facial point mapping and prosody analysis in speech. Subsequently, the federated model was trained locally on devices such as smartphones, tablets, and laptops, using federated averaging algorithms to combine the model updates on a central server without compromising data privacy (Doriguzzi-Corin and Siracusa, 2024).

The results of this approach show that the proposed model achieves competitive metrics in terms of precision 87%, recall 85%, and F1-score 86%, which positions it as a robust alternative to centralized systems such as DeepFace and commercial solutions such as Affectiva SDK (Hammann et al., 2022). Furthermore, robust tests performed under adverse conditions, such as variations in lighting and environmental noise, demonstrated that the model maintains consistent performance, outperforming centralized models in similar scenarios. For example, the model’s precision in low lighting conditions was 80%, compared to 75% for centralized models evaluated under the same conditions.

Integrating the model into Moodle, a widely used learning management system, enabled us to evaluate its practical applicability in a conventional educational environment (Shchedrina et al., 2021). This process demonstrated the system’s ease of adoption and highlighted its positive impact on student behavior. The results indicate that positive emotions, such as motivation, detected by the system are associated with a 15% increase in academic engagement and a 12% improvement in students’ average academic performance. In contrast, although it is more challenging to detect negative emotions, such as stress and frustration, it provides valuable data to adjust educational strategies and provide targeted emotional support.

Despite these advances, the model faces limitations inherent to the federated approach, such as dependence on heterogeneous devices and sensitivity to the quality of network connections during the model aggregation process. Although significant, these limitations do not compromise the system’s viability; instead, they highlight the need for future research to optimize its performance in environments with limited technological infrastructure.

This study’s main contribution lies in combining privacy, scalability, and performance in an emotion detection system specifically designed for educational environments. It aims to determine the extent to which a federated learning model can accurately identify students’ emotional states, both explicit and nuanced, using data from fundamental academic interactions across multiple modalities. The work further explores how decentralized training affects model reliability under real-world constraints, including limited infrastructure and diverse emotional expression patterns. Unlike existing solutions, the proposed approach ensures the confidentiality of emotional data while providing an adaptable and practical tool for academic institutions.

The remainder of this article is structured as follows: Section 2 presents a literature review on emotion detection systems, highlighting the current limitations in terms of privacy and scalability. Section 3 describes the materials and methods, including the data collection process, preprocessing techniques, and the design of the federated learning architecture. Section 4 presents the experimental results, including performance comparisons, robustness evaluations, and assessments of real-world impact. Section 5 discusses the findings about existing literature, addresses limitations, and outlines future research directions. Finally, Section 6 summarizes the main contributions and conclusions of the study.

## 2 Literature review

Emotion detection has been the subject of numerous studies examining various approaches to identifying human emotions in diverse contexts. Among these approaches, centralized systems such as DeepFace (An et al., 2023) have demonstrated high performance in emotion classification based on facial features (Anand and Babu, 2024). DeepFace uses highly trained convolutional neural networks (CNNs) to process images on central servers (Zhang Y. et al., 2024), achieving precision levels of up to 90% in emotion detection tasks. However, this centralized model faces significant criticism due to the need to transfer sensitive personal data to external servers, compromising user privacy. This aspect limits its applicability in educational settings, where data protection is a priority.

Another prominent approach is hybrid transformer-based models, such as those presented by Teng et al. (2024), which combines image, audio, and text processing to achieve more robust emotion detection. These systems can analyze multiple modalities, integrating contextual and temporal features into their architecture. Although transformers offer advantages in terms of flexibility and performance, their high computational cost and reliance on large volumes of data limit their deployment in resource-constrained educational settings. Moreover, like centralized systems, these models often require transferring data to external servers, posing similar risks to privacy.

Commercial systems, such as the Affectiva SDK, have been specifically designed for practical applications in marketing and behavioral analysis (Kulke et al., 2020). This software utilizes advanced computer vision techniques to identify facial emotions in real-time and is optimized for commercial platforms. Affectiva stands out for its ease of use and competitive performance (Shwe Sin and Khin, 2022), with accuracies ranging from 85 to 88%. However, its closed approach and high licensing costs hinder its adoption in educational settings, where budgets are often limited, and custom configurations are essential to integrate into existing platforms such as Moodle.

Federated learning emerges as an innovative solution to address the privacy and scalability limitations of centralized models. Huang et al. (2023) demonstrated the potential of federated architectures for emotion detection; however, their framework exhibited limitations in device heterogeneity, requiring uniform client capabilities and stable communication channels. These constraints limited scalability and reduced effectiveness in dynamic educational environments, where device resources and connectivity vary considerably. Moreover, their work did not include practical integration with learning platforms, which limited its pedagogical impact and real-time applicability within classroom systems.

Compared to the reviewed models, the federated approach proposed in this work stands out for its ability to balance performance, privacy, and scalability. It explicitly addresses the technical challenges noted by Huang et al. (2023) by introducing adaptive preprocessing techniques that tolerate device variability, optimizing local training for constrained devices, and integrating directly with Moodle and other learning management platforms. This reduces deployment complexity and supports institutions with limited infrastructure (Mukta et al., 2024). Despite advances in federated learning, existing literature has yet to explore its comprehensive implementation in hybrid academic environments that combine real users, platform integration, and privacy-by-design principles. This work seeks to address this shortcoming by presenting an integrated and deployable system designed for emotion detection in educational settings.

## 3 Materials and methods

### 3.1 Description of the test environment

The federated learning-based emotion detection system was implemented in a university educational environment, specifically in the Faculty of Technologies, which includes approximately 650 students. This environment is characterized by a hybrid education modality, meaning that students attend classes both in person and online. This hybrid modality presents an interesting challenge for implementing emotion detection technologies, as students interact with content and teachers in multiple ways—either in the physical classroom or through digital platforms—enabling the collection of emotional data in diverse contexts.

The Faculty of Technologies offers training programs in disciplines related to computer science, electronic engineering, and communication networks. This academic profile makes the federated learning approach particularly suitable, as most students are familiar with using technological tools and are active users of smart devices, which facilitates the adoption of the proposed technology for emotion detection.

A total of 150 students were selected to participate in the study, representing approximately 23% of the faculty’s total student population. This group was chosen randomly but representatively, ensuring the inclusion of students from different majors within the faculty and capturing a diverse sample of emotions. In addition, 20 teachers actively participated in the study, allowing for the monitoring of students’ emotional well-being throughout the course, both in face-to-face and online classes.

The selected sample consisted of undergraduate students with an average age of 21.2 years (SD = 1.7), ranging from 18 to 25. Gender distribution was approximately 56% male and 44% female. Students came from three main academic programs: Computer Science, Electronic Engineering, and Communication Networks. All participants were enrolled in hybrid courses that combined in-person and virtual components, ensuring a wide range of interaction modalities with the system. This diversity supports the generalizability and robustness of the experimental findings.

The implementation occurs in an online and hybrid education environment, providing an ideal opportunity for collecting emotional data in real-time and asynchronous interactions (Pirrone et al., 2021). Students interact with the system through various devices, either during online classes, remote exams, or discussion forums and activities within the Moodle platform, which served as the Learning Management System (LMS) in this pilot test.

The emotional data collection process is performed through various smart devices, such as smartphones, tablets, and laptops, which are standard in the faculty and integrated into the students’ daily activities. These devices capture emotional data through facial expression analysis, emotion detection through tone of voice during oral interactions, and text analysis in written responses on LMS platforms, mainly in forum activities and assessment tasks.

Each device acts as a node in the federated system, where the emotional data captured on each one is processed locally to preserve the privacy of the students (Ribeiro Junior and Kamienski, 2024). The students’ devices preprocess the emotional data through applications developed specifically for this test, extracting relevant features from facial images, vocal tone, and textual responses. The emotion detection model is trained locally on these devices, using the data collected in real-time, without such data leaving the device (Almalki et al., 2024).

Students can participate in the system through the mobile app and on their desktop devices without requiring constant direct interaction with the system, thus allowing the federated learning model to adapt to variations in emotions throughout the educational day. Teachers can access the emotion reports generated without compromising students’ privacy and use these reports to adjust their pedagogical strategies in real time, especially regarding student workload and stress during classes and assessments.

The system infrastructure is based on federated architecture, where student devices train the emotion detection model independently. Communication between the devices and the central server is limited to model updates only, ensuring that sensitive data is not shared at any time (Zhou et al., 2024)Model updating employs techniques such as federated averaging, which enables the central server to aggregate updates to local models without requiring the original data for each student to be centralized.

**Figure 1:**
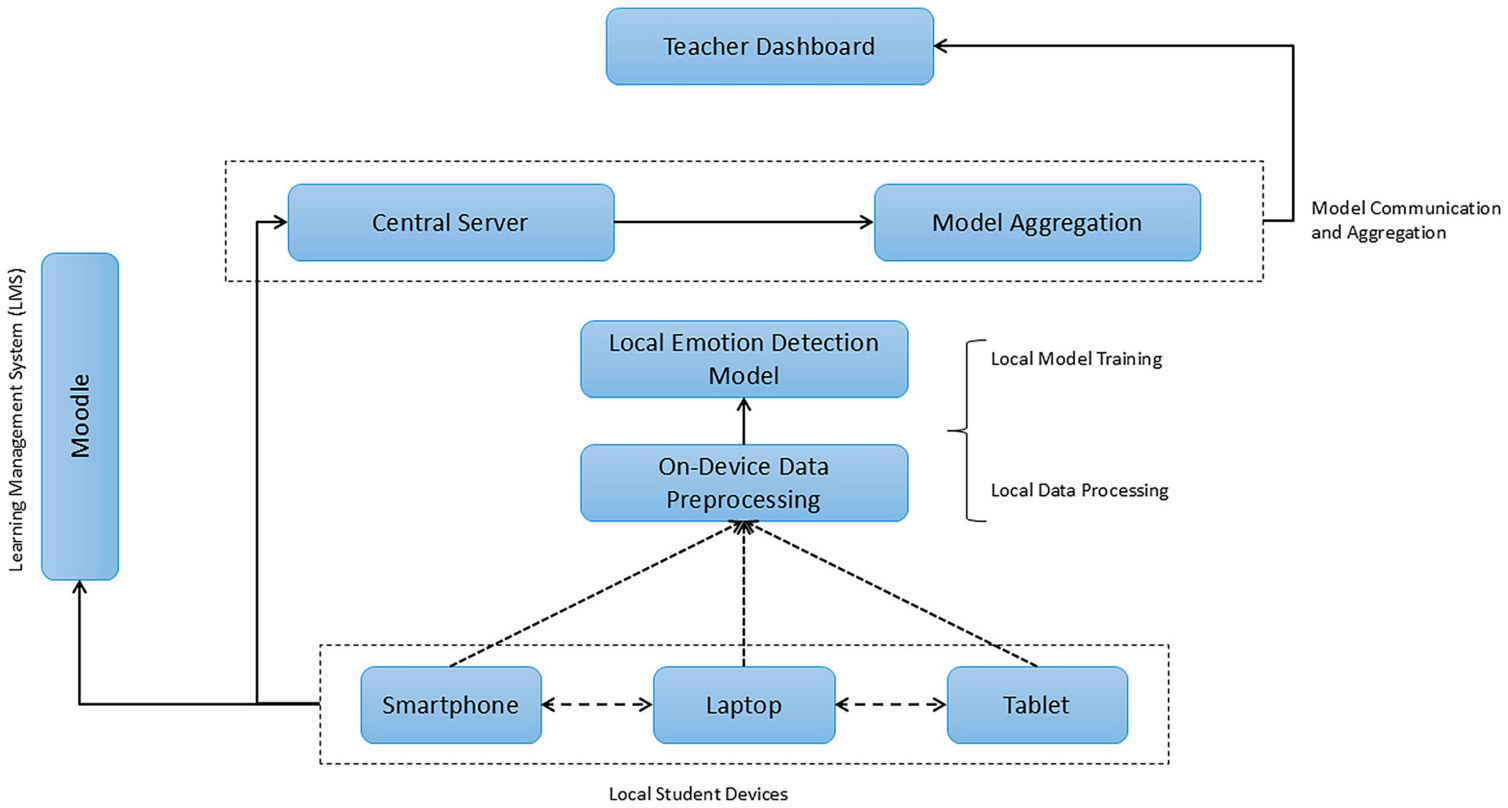
Architecture of the federated learning-based emotion detection system.

Figure 1 presents the architecture of the proposed system for emotion detection using federated learning. It demonstrates how local student devices, such as smartphones, laptops, and tablets, interact with the system. Each device performs local preprocessing of the emotional data, extracting relevant features from facial expressions, tone of voice, and textual interactions. Once the regional models are trained on the devices, the model updates are sent to the central server for aggregation through a federated averaging process. This process allows the central server to combine the model updates without centralizing the original student data, ensuring data privacy (Zhang H. et al., 2024). Furthermore, aggregated reports of student emotions are visualized through the Teacher Dashboard, providing teachers with valuable information about the class’s emotional well-being without requiring access to individual personal data. The connection to Moodle enables the capture of students’ academic opinions in real-time, while model updates continually improve as more emotional data is collected.

### 3.2 Data collection

#### 3.2.1 Emotion capture method

Three specific techniques are used for emotion detection: facial expression analysis, voice tone detection, and text analysis. These techniques are applied complementarily to ensure a complete and accurate assessment of students’ emotional states during educational interactions. Facial expression analysis is based on the premise that human emotions are reliably reflected in facial movements, which are detected and classified with high precision using computer vision techniques (Lyu, 2023). This process is carried out using a CNN-based model, which allows the identification of key facial features such as eye, mouth, and eyebrow movements. Through the front-facing cameras of the devices, the system captures the students’ facial expressions in real-time. The data obtained is processed locally on each device to extract the relevant emotional features, allowing the detection of emotions such as happiness, sadness, anger, surprise, contempt, and disgust, which correspond to the basic emotions identified by Ekman et al. (1998). The application of this model is carried out continuously during the student’s interactions with the academic environment, ensuring the accurate capture of emotions in various situations. However, it is acknowledged that some of these interactions may occur outside the core academic platform (e.g., Moodle), were external, non-educational factors could influence emotional variation. These factors lie beyond the teacher’s control and could introduce biases in interpreting students’ emotional states, a limitation also highlighted in recent ethical studies on emotion recognition in educational settings (Di Dario et al., 2024).

Voice pitch detection is another crucial method in emotion detection. This process involves capturing and analyzing variations in the acoustic features of the voice, such as fundamental frequency, intensity, duration, and prosody (Jiang et al., 2023). These features indicate emotional variations in speech, as vocal pitch and rhythm change in response to the individual’s emotional state. Microphones in the devices pick up the student’s voice during oral interactions. Using audio signal processing algorithms, such as acoustic feature analysis and time-sequence modeling, variations in speech are analyzed to identify emotions, including stress, confusion, or satisfaction. A model based on Recurrent Neural Networks (RNN), specifically Long Short-Term Memory (LSTM), is used, which can identify emotional patterns throughout voice sequences, allowing accurate detection in dynamic situations (Chen et al., 2017).

Text analysis examines the emotional content of students’ written responses on platforms such as Moodle, especially in forum interactions, assignments, and exams. The system identifies linguistic patterns related to emotions using natural language processing (NLP) techniques (Merity et al., 2018). Advanced models, such as Bidirectional Encoder Representations from Transformers (BERT), are applied, which can analyze the semantic context of words and phrases within texts. This analysis allows the classification of underlying emotions, such as anxiety, motivation, or confusion, from written interactions (Subakti et al., 2022). Text data is processed in real time, assessing students’ emotions based on their written expressions during academic activities.

#### 3.2.2 Devices and sensors

Emotional data is collected using various devices and sensors embedded in students’ devices, specifically smartphones, tablets, and laptops. Each device has specific technologies that accurately capture emotional data based on the interaction modality.

The cameras capture facial expressions, enabling the real-time visual analysis of emotions. The cameras can identify and classify facial patterns related to basic emotions, such as happiness, sadness, anger, surprise, and others.

On the other hand, the microphones built into the devices allow for capturing the acoustic characteristics necessary to analyze voice tone. These microphones are designed to capture sounds at an appropriate frequency, enabling the detection of variations in pitch, volume, and speech rate that indicate different emotional states. Through these microphones, the system can identify whether the student is experiencing emotions such as stress or satisfaction, which correlates with the tone and dynamics of their voice.

The Moodle platform is used for collecting textual data. Students interact on the platform through forums, assignments, and exams, generating written responses that are then processed to assess the underlying emotions in their content. The system analyzes the words, phrases, and text structure using natural language processing models to identify emotional states related to the content of the responses, such as anxiety, motivation, or confusion. Table 1 summarizes the devices, sensors, and platforms employed, along with their respective functions in the emotional data collection process.

**Table 1:** Devices, sensors, and platforms used for collecting emotional data.

Device/sensor	Function	Associated technique
Smartphone/tablet/laptop	Capture of emotional data through a camera and a microphone	Facial analysis, voice detection, text analysis
Camera	Capture of facial expressions for real-time analysis	Facial expression analysis
Microphone	Capture of tone of voice, variations in frequency, and amplitude for emotion detection	Voice tone analysis
Moodle (LMS)	A platform for collecting textual responses through educational interactions	Text analysis

All devices used for data collection were the personal property of the students. The emotion detection system was not pre-installed; instead, it was accessed entirely through the Moodle Learning platform, which provided a seamless interface for data capture and analysis. This integration ensured that no additional software needed to be installed on student devices, thereby minimizing intrusiveness and maintaining user autonomy. Additionally, the system’s architecture ensures that all data is processed locally on the device, aligning with the principles of privacy-by-design.

### 3.3 Data preprocessing

In the filtering and anonymization process, specific techniques are applied to protect sensitive data, especially students’ facial and audio features (Hanisch et al., 2024). Facial expression data is processed to remove backgrounds and lighting variations irrelevant to emotion detection. This filtering is performed by a face segmentation algorithm using the OpenCV library, which detects the exact location of the face within the image and crops only the region of interest. A Gaussian smoothing filter is applied to the face region to reduce background noise and ensure that only relevant facial features are processed (Nandan et al., 2024).

Data anonymization is performed by modifying the detected facial points so the individual cannot be identified. In the case of facial analysis, key landmarks, such as the eyes, eyebrows, and mouth, are replaced by generic points that do not correspond to a specific identity. This technique uses facial mapping algorithms that randomly relocate facial features within a range of standard facial parameters (Wang, 2024). In addition, the data is not stored in its original form; instead, only the model updates are sent, implying that the facial images never leave the local devices and do not contain identifiable information.

In encryption, the Advanced Encryption Standard (AES-256) encrypts the model parameters when they are sent from the local devices to the central server (Ajagbe et al., 2024; Mishra et al., 2024). This encryption ensures that even if the data is intercepted, it cannot be decrypted without the proper key, protecting the students’ privacy during model communication. The feature extraction process for each data type (facial expressions, voice, and text) is carried out using specific algorithms designed for each modality.

To ensure clarity and reproducibility, the emotional states targeted by each modality were explicitly defined and consistently applied throughout the training and evaluation phases. Each modality was associated with a distinct subset of emotional labels based on the nature of the data and the capabilities of the corresponding model. These labels were selected from well-established emotional taxonomies that have been adapted for educational settings. Table 2 summarizes the exact emotions detected by facial expressions, voice signals, and textual content.

**Table 2:** Emotional states detected by each modality.

Modality	Emotional states detected
Facial expression	Happiness, sadness, anger, surprise, disgust, contempt
Voice (audio)	Stress, confusion, satisfaction, boredom, engagement
Textual content	Motivation, anxiety, confusion, frustration, curiosity

#### 3.3.1 Facial expression detection

In facial expression analysis, Dlib’s facial point detection algorithm identifies critical points on the face, such as the contours of the eyes, nose, and mouth. The mathematical process underlying this algorithm is based on supervised learning and nonlinear regression techniques. Once these points are detected, the Active Shape Model (ASM) is used to model the variability in the shape of the face (Alavi et al., 2024).

The ASM can be described mathematically by an elastic deformation model that adjusts parameters to capture facial variation. The warping algorithm uses affine transformation matrices, where each transformation *T* is represented by a parameter matrix *Ɵ* that adjusts the position of each facial point *p(x, y)* in the image according to the warping model of the Equation (1):


p′(x,y)=T(θ)⋅p(x,y) (1)

*T* is the transformation matrix, which describes how facial points are adjusted according to emotional variations. In addition, the Euclidean distance between the detected facial points is calculated to measure the degree of change in facial expressions as shown in Equation (2):


$$\;d=\sqrt{{\left(x_{2}-x_{1}\right)}^{2}+{\left(y_{2}-y_{1}\right)}^{2}}$$ (2)

This allows the quantification of deformation in the face related to emotions such as surprise or sadness.

Speech signal analysis is based on acoustic features such as the fundamental frequency *(F_0_)* extracted using the Fast Fourier Transform (FFT). Mathematically, the FFT decomposes a signal *x(t)* into its frequency components, representing the signal in the frequency domain as a sum of sinusoids, as shown in Equation (3):


$$X(f)=\int_{-\infty }^{\infty }x(t)e^{-j2\mathrm{\pi ft}}\mathrm{dt}\;$$ (3)

*X*(*f*) is the frequency domain representation of the signal, *f* is the frequency, and *x*(*t*) is the time domain signal.

The fundamental frequency *(F_0_)* is the lowest component of the audio signal and is related to the pitch of the voice. This parameter is extracted to measure emotional variations in the voice pitch, such as when anger or joy is detected. Additionally, prosody analysis is employed, which examines the intensity and rhythm of the voice. Mathematically, rhythm can be measured in terms of syllable duration and speech rate, and intensity is evaluated as the amplitude of the audio signal in each time window using energy measurement formulas, as shown in Equation (4):


$$E(t)=\sum_{n=0}^{N}|x(t+n)|2\;$$ (4)

where *E*(*t*) is the energy in a time window, *x*(*t* + *n*) is the value of the audio signal at time *t + n*, and *N* is the number of samples within the time window.

Prosody analysis is then used to feed the LSTM model, which applies backpropagation through time (BPTT) to update the neural network weights and model emotions based on speech’s pitch and temporal variability. LSTMs use activation functions such as sigmoid or tanh, which classify emotions based on the temporal content of the signal.

#### 3.3.2 Text analysis

Text analytics is based on transformer models, such as BERT, designed to capture the bidirectional context of words within a sentence (Kotwal et al., 2022). Mathematically, this model is a word embedding, which maps words to high-dimensional vectors in a vector space, using functions such as softmax to generate classification probabilities.

In mathematical terms, the embedding process is described by a projection of each word *w_i_* into a *d*-dimensional feature space, as shown in Equation (5):


$$v_{i}=f(w_{i})\;$$ (5)

where *v_i_* is the feature vector of the word *w_i_*, and *f* is the projection function learned during training.

Using self-attention, the BERT model captures contextual relationships between words, which computes the weighted relationship between words within a given context. Attention is mathematically defined in Equation (6):


$$\;\text{Attention}\;(Q,K,V)=\text{softmax}(\frac{QK^{T}}{\sqrt{d_{k}}})V\;$$ (6)

where *Q, K,* and *V* are the query, key, and value matrices, respectively, and *d_k_* is the dimension of the keys. This attention mechanism enables the model to capture long-range dependencies within the text, allowing it to detect complex emotions such as frustration or motivation.

Once the vector representations of the words are obtained, they are used to classify the emotions associated with the text through a deep neural network that adjusts the weights using the backpropagation algorithm and the softmax activation function to obtain the probability of each emotion, as shown in Equation (7):


$$P\;({\text{emotion}}_{i})=\frac{e^{z_{i}}}{\sum_{j}e^{z_{j}}}\;$$ (7)

where *z_i_* are the network outputs for each emotional class, and *P*(*emotion_i_*) is the probability that the emotion is present in the text.

#### 3.3.3 Model training by modality and dataset description

For emotion detection in educational contexts, three specialized models were developed, each adapted to a different modality: facial images, voice signals, and written text. These models were trained using public datasets widely validated in the literature, ensuring their availability and validity for emotion classification tasks. Furthermore, invasive collection processes or those dependent on sensitive information were avoided, aligning with the privacy principles defined in the overall system design.

For emotion detection using facial expressions, the JAFFE dataset was utilized, which comprises 213 images with a resolution of 48 × 48 pixels, categorized into seven emotional states: happiness, sadness, anger, surprise, fear, disgust, and neutrality. These images were captured in uncontrolled scenarios, enabling the model to generalize more effectively in real-life conditions. For speech-based detection, the Ryerson Audio-Visual Database of Emotional Speech and Song (RAVDESS) dataset was used. It consists of 1,440 audio clips recorded by professional actors expressing eight emotions (calm, happiness, sadness, anger, fear, disgust, surprise, and neutral). Finally, for the textual modality, the EmotionX dataset, which focuses on fundamental conversational interactions, was utilized. This corpus includes brief responses manually labeled with emotions such as happiness, sadness, anger, motivation, frustration, or surprise. All datasets were openly accessible, and no private data was included; additionally, no manual labeling was performed.

Each model was designed to respond to the specific characteristics of its modality. The facial image model employed a VGG-13-based architecture, comprising two convolution blocks with 64 and 128 filters, respectively, followed by max pooling operations and dense layers of 512 and 128 units, before the softmax output layer. ReLU activation functions and a dropout value of 0.5 were used to prevent overfitting. For speech modality, the model was built on an LSTM network with 256 hidden units, followed by a dense layer with 64 neurons and a softmax output tuned to eight classes. The input consisted of sequences of MFCC coefficients extracted from 25-ms segments. Batch normalization, a dropout of 0.3, and the categorical cross-entropy loss function were employed. For text, the BERT model (uncased version, 110 million parameters) was implemented, to which a dense layer with 128 neurons and a softmax output of six classes was added. Fine-tuning was performed only on the last four layers of the transformer to preserve the pre-trained semantic capacity.

The three models were trained using a familiar hyperparameter setting, with slight variations tailored to the computational needs of each modality. A batch size of 32 was used for images and speech, and 16 for text. The initial learning rate was 0.0001, with the Adam optimizer and a weight decay penalty of 1e-5. The maximum number of epochs was set to 50, with an early stopping mechanism activated if no improvement was observed in validation after 10 iterations. In all cases, the sets were divided into 70% for training, 15% for validation, and 15% for testing, following a consistent protocol across modalities.

The training environment consisted of notebooks developed in Python 3.9 using PyTorch 2.0, HuggingFace Transformers, and the librosa library for acoustic feature extraction. The experiments were conducted on Google Colab Pro+ with access to a 16 GB Tesla T4 GPU and 52 GB of RAM, enabling efficient and reproducible training. It is essential to clarify that these models were not trained directly on student data, but rather pre-trained on the datasets above and subsequently deployed in a federated architecture. The federated process involved three to five local fine-tuning cycles per device, enabling the models to gradually specialize according to the emotional characteristics of the real-life educational environment, while preserving user privacy.

To complement these public datasets, the models were not deployed in their pre-trained form only. Once integrated into the federated environment, each modality was fine-tuned locally using anonymized records derived from fundamental student interactions within the Moodle platform, including forum messages, voice participation, and facial expressions captured during hybrid sessions. This local fine-tuning process ensured that the models adapted to the specific linguistic, acoustic, and behavioral characteristics of the target educational population, while respecting privacy constraints. Importantly, no raw interaction data was centralized; only model updates were transmitted following the principles of federated learning. In this way, the training strategy combined the robustness of publicly validated datasets with the contextual specificity of real-world data, ensuring methodological consistency and ecological validity.

To address the mismatch between the target emotional categories and the labels present in the pre-training corpora, additional open datasets and a harmonization strategy were incorporated. For facial modality, supplementary corpora such as AffectNet (Mollahosseini et al., 2019) were used to include classes not covered by FER2013 (Santoso and Kusuma, 2022), particularly contempt, while still relying on FER2013 as the baseline for basic facial emotions. In the audio modality, RAVDESS was expanded with resources like EMO-DB, IEMOCAP, and RECOLA (Joudeh et al., 2023; Khurana et al., 2024; Ong et al., 2024), which provide categories closely aligned with stress, confusion, and boredom. Prosodic dimensions from these corpora, mapped along valence–arousal axes, enabled the derivation of satisfaction and engagement-related cues. For text, datasets such as GoEmotions and education-specific corpora were integrated, ensuring coverage of states like motivation, anxiety, and curiosity through semantic mapping and weak supervision techniques (Demszky et al., 2020).

This process followed a label-space harmonization approach in which semantically equivalent or proximate categories from different sources were merged into a unified taxonomy. Mapping was supported by distributional similarity measures and embedding-based alignment to maintain consistency across modalities. When labels were absent from the pre-training corpora but present in supplemental ones, transfer learning mechanisms were employed to transfer representations into the federated fine-tuning stage.

Regarding FER2013, only the 32,298 publicly available images were used, as the remaining portion of the original corpus is restricted and inaccessible. The dataset is distributed into a predefined training split of 28,709 images and a test split of 3,589 images. To introduce a validation stage consistent with the 70/15/15 strategy applied across modalities, we further partitioned the training split by reallocating 15% of its samples (≈4,307 images) as a validation subset, while retaining 24,402 images for training. The original test split of 3,589 images was preserved without modification to serve as the final evaluation set. This procedure ensured methodological uniformity across modalities while maintaining compatibility with the canonical FER2013 evaluation protocol, thereby avoiding the use of non-public data and reinforcing the reproducibility of the experiments.

Finally, specific high-level affective constructs, such as engagement, were not directly predicted by a single classifier but inferred through multimodal fusion. In these cases, the system combined audio-prosodic indicators, facial activation levels, and behavioral traces from LMS interactions to derive a composite state. This ensured that all emotional categories defined in the study were technically grounded, either through explicit dataset coverage, mapped proxies, or composite modeling strategies aligned with the federated architecture.

#### 3.3.4 Definition of emotion classes by modality

The definition and categorization of the emotional states targeted in this study were established to guarantee clarity, reproducibility, and technical rigor. Each modality—facial expressions, voice signals, and textual content—was associated with a set of emotional labels aligned with validated taxonomies in affective computing and educational psychology. This structured definition ensured that the classification tasks were consistent across modalities and that the evaluation of the federated system was traceable and comparable with existing models.

For facial modality, the taxonomy proposed by Ekman and Rosenberg was adopted, as it provides a robust foundation for identifying emotions that are consistently expressed through facial movements (Ekman et al., 1998). Emotions such as happiness, sadness, anger, surprise, contempt, and disgust were selected because they present distinctive visual cues that can be quantified using convolutional neural networks. These categories have been repeatedly validated in emotion recognition studies, allowing for a reliable mapping between observable facial features and underlying affective states.

In the voice modality, the classes of stress, confusion, and satisfaction were defined, given their strong correlation with variations in prosodic features such as pitch, intensity, and rhythm. These emotions are adequately represented in corpora like RAVDESS and are critical in educational contexts, where vocal modulation often reflects cognitive load and affective responses to learning tasks. By focusing on these specific states, the system captures meaningful indicators of students’ emotional dynamics in oral interactions (Bilotti et al., 2024).

In addition to stress, confusion, and satisfaction, the model also incorporated two derived affective states—boredom and engagement. These states were not directly annotated in the base RAVDESS corpus. Still, they were obtained through the integration of IEMOCAP and RECOLA datasets, where prosodic patterns were mapped along the valence–arousal plane. Boredom was associated with low arousal and neutral-to-negative valence speech segments, while engagement corresponded to high arousal and positive valence prosodic patterns. These derived states were incorporated through label harmonization and validated during the federated fine-tuning phase, allowing the model to infer motivational intensity from voice cues.

In the textual modality, emotions such as anxiety, motivation, and frustration were prioritized. These categories are highly relevant in written academic interactions, where students frequently express their affective states indirectly through language. Using transformer-based semantic embeddings, particularly BERT, the system was able to analyze the bidirectional context of written responses, capturing subtle variations in meaning that reflect students’ affective conditions (Sayeed et al., 2023). Beyond motivation, anxiety, and frustration, two additional affective states—curiosity and confusion—were integrated through semantic mapping using the GoEmotions corpus and education-specific text samples. Curiosity was identified through linguistic constructions reflecting positive exploratory intent (e.g., interrogative forms combined with positive sentiment). In contrast, confusion emerged as a composite category derived from frustration and uncertainty labels through weak supervision. These categories were retained during fine-tuning as they frequently occur in learning contexts, enabling more accurate modeling of cognitive-affective dynamics in student writing. The taxonomy, organized by modality, aligns with Table 2, where basic emotions (e.g., happiness, sadness) coexist with derived and context-specific states (e.g., engagement, curiosity).

This threefold definition of emotional classes provides a rigorous framework for the federated model, ensuring that each modality contributes in a complementary manner to the global detection process. The careful alignment of modalities with distinct emotional categories avoids overlaps, reduces ambiguity in classification, and reinforces the interpretability of the results obtained in world educational environments.

### 3.4 Development of the emotion detection model

#### 3.4.1 Emotion detection models

Different types of deep learning models are used to address emotion detection in students, tailored to the specific characteristics of each data modality: facial images, audio, and text. These models have been selected for their ability to learn complex, high-level representations of emotional data, and each one specializes in the type of data it is provided with.

First, CNNs are employed for facial expression analysis, which can extract spatial features from facial images. CNNs are especially effective in computer vision tasks due to their ability to identify hierarchical patterns of information, ranging from simple features such as edges and textures to complex patterns, including emotions expressed on the face. The model is trained using high-resolution facial images, where the network learns to identify spatial relationships between key points on the face.

CNNs operate by applying convolutional filters to images, where each filter *W_k_* generates a feature map *C_k_* as defined in Equation (8):


$$\;C_{k}=W_{k}*I\;$$ (8)

where *I* is the input image and * denotes the convolution operation. These feature maps are combined to extract emotions such as happiness, sadness, anger, or surprise.

An RNN and an LSTM are used for voice tone analysis and are ideal for processing temporal data sequences such as audio signals. LSTMs are designed to capture long-term dependencies in audio sequences, which is crucial for identifying emotions that evolve in a conversation or speech (Hashmi and Yayilgan, 2024). LSTM parameters, such as input, output, and forget gates, allow the network to remember and forget information based on the temporal characteristics of the signal. Mathematically, the LSTM model is defined by the following Equations (9)– (13):


$$\;f_{t}=\sigma (W_{f}\cdot [h_{t-1},X_{t}]+b_{f})$$ (9)

$$\;i_{t}=\sigma (W_{i}\cdot [h_{t-1},X_{t}]+b_{i})$$ (10)

$${\hat{C}}_{t}=\tan h(W_{c}\cdot [h_{t-1},X_{t}]+b_{c})$$ (11)

$$C_{t}=f_{t}*C_{t-1}+i_{t}*{\hat{c}}_{t}$$ (12)

$$h_{t}=o_{t}*\tanh(C_{t})$$ (13)

where *f_t_*, *i_t_*, and *o_t_* are the forget, input, and output gates, *x_t_* is the temporal input (audio signal), *h_t_* is the hidden state, and *C_t_* is the cell state.

A transformer-based model, specifically BERT, is used for text analysis, which is highly effective at processing text bidirectionally. The BERT model can understand the full context of a word within a sentence, as it examines both the preceding and following contexts of the word. This approach outperforms traditional one-way models and is particularly useful for understanding complex emotions in language. Text analysis in BERT is done through word embedding, where each word is transformed into a high-dimensional vector that captures its context.

#### 3.4.2 Federated model

The federated learning model implemented in this study enables emotion detection models to be trained in a decentralized manner, i.e., without centralizing sensitive data on a server. This approach is crucial for ensuring student privacy, as only model updates are shared, not the original data.

Each student device uses data to train the emotion detection model during local training. This process is conducted locally, meaning that each student’s emotional data remains on their device. The model on each device is continuously tuned and improved as more emotional data is collected from the student’s interactions with educational content.

The local model performs parameter updates using the gradient descent algorithm. Since the data is not centralized, training is carried out in parallel on each device without sharing information about the students’ data. The model parameters, which are weight vectors *w_i_*, are updated based on the local error calculated at each device, and the update follows the standard gradient rule, as expressed in Equation (14):


$$\;w_{i}=w_{i}-\eta \cdot \nabla_{w_{i}}L$$ (14)

where η is the learning rate, and $\nabla_{w_{i}}L$ is the gradient of the loss function *L* concerning the parameters *w_i_*.

To integrate emotional information obtained from the three data modalities, facial images, voice recordings, and text inputs, the system employs a late fusion strategy. Each modality is processed independently on the student’s device using the respective specialized models: a CNN for facial expressions, an LSTM for voice tone, and a transformer (BERT) for textual data. Each model outputs a probability distribution over the predefined set of emotional classes. These three distributions are then combined using a weighted average, where the weights were empirically tuned during the development phase to optimize overall classification performance. The final emotional prediction corresponds to the class with the highest combined probability. This modular approach enables flexible processing even in scenarios where one or more modalities are temporarily unavailable (e.g., no audio input), ensuring the robustness and adaptability of the federated learning system.

#### 3.4.3 Model aggregation

Once the local model has been trained on each device, the model parameter updates are sent to the central server, which combines them using an aggregation process. In federate learning, this is done using the federated averaging algorithm. This method allows the central server to combine model updates without accessing the original learner data (Ren et al., 2024).

Mathematically, federated averaging can be expressed as a weighted average of the local model updates, denoted as the local model updates $\Delta_{w_{i}}$ from each device *i* as shown in Equation (15):


$$\mathrm{\Delta w}=\frac{1}{N}\sum_{i=1}^{N}{\mathrm{\Delta w}}_{i}$$ (15)

where *N* is the total number of devices participating in the training, the central server calculates the weighted average of the parameter updates ${\mathrm{\Delta w}}_{i}$ and fine-tunes the global model, which is then distributed back to the devices to continue the training process.

This local training and federated aggregation process enables continuous improvement of the emotion detection model without requiring centralization of data. It ensures that learners’ privacy is preserved while the model continues to learn collectively. The result is a more accurate and robust global model that can detect emotions in real-time, with sensitive data never shared outside local devices.

### 3.5 Evaluating model precision

Several standard metrics are used in machine learning to evaluate the performance of the emotion detection model. These metrics are essential for understanding how the model identifies emotions, both in terms of precision and recall, and for gaining a comprehensive view of its performance. The metrics used in this study are as follows.

Precision: Precision measures the proportion of correct predictions of a positive class (e.g., the “happy” emotion) among all projections of that class. Mathematically, it is expressed as:

Precision is defined as shown in Equation (16):


$$\;\text{Precision}=\frac{\mathrm{TP}}{\mathrm{TP}+\mathrm{FP}}$$ (16)

where:

- *TP* (True Positives) are the correct predictions of the positive class.
- *FP* (False Positives) are the incorrect predictions of the positive class.

Recall measures the ability of the model to detect all positive instances (specific emotions) in the data. It is calculated as shown in Equation (17):


$$\;\text{Recall}=\frac{\mathrm{TP}}{\mathrm{TP}+\mathrm{FN}}$$ (17)

where:

- *FN* (False Negatives) are the positive instances that the model incorrectly classified as negative.

Accuracy measures the proportion of correct predictions among all predictions made. It is a general metric that indicates how well the model classifies all emotions, as shown in Equation (18):


$$\;\text{Accuracy}=\frac{\mathrm{TP}+\mathrm{TN}}{\mathrm{TP}+\mathrm{FP}+\mathrm{TN}+\mathrm{FN}}$$ (18)

where:

- *TN* (True Negatives) are the correct predictions of the negative classes.

The F1-Score is a metric that combines precision and recall into a single figure, making it useful when seeking a balance between the two. It is calculated as the harmonic meaning between precision and recall, as shown in Equation (19):


$$\;F1-\text{score}=2\cdot \frac{\text{Precision}\cdot \text{Recall}}{\text{Precision}+\text{Recall}}$$ (19)

The F1-score is particularly important in contexts with an uneven distribution of emotional classes, such as when one emotion is much more represented than others. This metric is critical to evaluating how the model handles less frequent emotions.

In addition to the automated evaluation, a manual validation process was conducted on a stratified sample of student responses across emotion categories. Annotators with expertise in affective computing assessed the alignment between the model’s predictions and the emotional intent expressed. This process confirmed high concordance with automated predictions in frequent emotions, such as happiness and sadness, while also revealing areas of potential ambiguity in less frequently represented categories, such as contempt or surprise. These findings reinforce the reliability of the system in real educational scenarios, especially when addressing common emotional states.

#### 3.5.1 Evaluation of the federated system

The federated model is evaluated in comparison to traditional emotion detection approaches that centralize data. The main advantage of the federated system lies in its ability to preserve students’ privacy while maintaining a high level of precision.

In a traditional emotion detection approach, all data (facial images, voice recordings, and text) are sent to a central server for model training. This approach poses risks of privacy violations, as sensitive data, such as expressed emotions, is stored and processed on a central server, increasing the chances of personal information being accessed without proper consent.

The federated model, in contrast, performs all processing and training locally on students’ devices. Only model parameter updates are sent to the central server, meaning emotional data is not centralized at any point (Pedrycz, 2023). Students’ privacy is kept intact, as no sensitive information is shared during training. Furthermore, the federated model presents a significant advantage in terms of scalability, as it enables model training to be performed in a distributed manner, utilizing the resources of each device without the need for centralized processing.

Regarding precision, the federated model has demonstrated comparable performance to traditional approaches, as updating model parameters on local devices follows a collaborative training process. Model updates performed by local devices are aggregated through federated averaging, allowing the global model to continue learning and improving collectively without compromising data privacy (Ren et al., 2023). The comparative evaluation between the federated model and the traditional approach, regarding precision and privacy, highlights that although federated models may face challenges related to device heterogeneity and variable local data quality, the privacy benefits and the ability to handle distributed data efficiently are decisive factors in their adoption.

The user-centered evaluation employed a validated instrument derived from the Trust between People and Automation scale (TPA) by Jian et al. (2000), which has recently undergone psychometric validation in the context of AI systems (Scharowski et al., 2025). The adapted version included 12 items covering dimensions of trust and distrust, aligned with factors such as perceived usefulness, reliability, and privacy confidence. Each item was rated on a 5-point Likert scale. This adaptation ensured methodological rigor and consistency with state-of-the-art approaches to AI trust measurement. A total of 42 students and six instructors participated in this evaluation. The responses were analyzed using descriptive statistics (meaning, standard deviation) and reliability analysis, which yielded a Cronbach’s alpha of 0.87, indicating high internal consistency. This quantitative assessment was complemented by short qualitative interviews with selected participants, providing further insight into system acceptance, concerns about data privacy, and perceived benefits in the learning environment. In addition to technical and privacy-related validation, the evaluation of the federated emotion detection system includes a user-centered study to assess perceived trust and usability, as well as an analysis of behavioral and academic changes observed after the system’s integration in real educational settings.

#### 3.5.2 Robustness testing

To assess the system’s robustness, tests are conducted under various conditions that may impact the model’s performance. These include environmental variations, such as changes in lighting for facial analysis, ambient noise in voice recordings, and emotional diversity among students:

- Variations in the Environment (Lighting Conditions and Ambient Noise): Lighting conditions can affect the quality of facial images, potentially hindering the precision of facial analysis. To mitigate this challenge, models are trained to be robust to changes in lighting using data augmentation techniques, such as adjusting brightness and contrast on facial images during preprocessing. Additionally, denoising filters are applied to the voice signals using techniques such as spectral analysis of the signal to minimize the impact of background noise.
- Diversity of Emotion: The diversity of emotions among students is assessed by ensuring that the model can correctly detect a wide range of emotions, from the most common ones, such as happiness or sadness, to less frequent emotions, such as surprise or confusion. Balanced datasets that include a broad emotional spectrum are used, and metrics such as the F1-Score are used to ensure that the model does not favor more prevalent emotions over others.

Generalization tests also evaluate the system’s robustness. The model is evaluated with data not seen during training to ensure it can make accurate predictions on new data, even from different lighting conditions, noise, or emotional contexts.

### 3.6 Ethical and privacy considerations

#### 3.6.1 Data protection

Protecting the collected data is essential to ensure students maintain their privacy throughout the process. Specific measures were implemented, including data anonymization, encryption of transmissions, and compliance with applicable privacy regulations. In the case of Ecuador, where the study was conducted, the system adheres to the principles established in the Ley Orgánica de Protección de Datos Personales (LOPDP). Additionally, international standards such as the General Data Protection Regulation (GDPR) were considered as a benchmark to ensure that the privacy protocols align with globally recognized practices (Hamon et al., 2022).

In terms of anonymization, it is ensured that facial data cannot be associated with a specific identity. During the collection of facial images, a facial blurring algorithm is used to process the images using a smoothing filter over the facial region (Hellmann et al., 2024). This filter erases specific facial features to maintain emotional expressions without compromising the student’s identity. To reinforce this anonymization, random facial mapping is applied, which shifts critical facial features, such as the eyes and mouth, to different locations in a controlled manner. This process ensures that facial information is not traceable to an individual student.

In addition to image anonymization, the data encryption process uses the AES-256 algorithm, which encrypts model updates before they are sent to central servers. This ensures that any transmitted data is protected and that even if it were intercepted, it could not be read without the proper key (Xu et al., 2024). Encryption is applied to model parameter updates, not the original data, meaning that sensitive data, such as the student’s emotions, never leaves the local device.

The system is designed to comply with relevant data protection regulations. In Ecuador, this includes adherence to the LOPDP. At the same time, international frameworks such as the European Union’s GDPR and the California Consumer Privacy Act (CCPA) served as reference models to guide best practices in data handling and transparency. All participating students were fully informed about the purpose and scope of the emotional data collection and provided their explicit consent before the acquisition of any data. Additionally, the study was reviewed and approved by the Research Ethics Committee of the University of Alicante, under the file number UA-2025-05-24, within the project titled “Gamificación educativa potenciada por inteligencia artificial.” This ethical approval ensures that the study adheres to institutional and international standards for research involving human participants, thereby reinforcing the protection of student rights, privacy, and autonomy.

#### 3.6.2 Transparency and consent

Transparency in emotional data is essential for students to feel comfortable participating in the system. Each student receives a clear and accessible document detailing how their emotional data is collected and processed. The system ensures that students have complete control over their data. Through an accessible interface, students can give explicit consent before participating in the system. This consent covers all aspects of emotional data processing and can be modified or withdrawn at any time. If a student withdraws consent, the system allows for the secure deletion of previously collected data, per current privacy regulations.

The right of students to withdraw consent at any time is fully respected. Once consent is withdrawn, the collected data is securely deleted through a data erasure process that ensures no trace of the information remains. This process complies with the regulations set by the GDPR and CCPA, which require deleting data when it is no longer needed or when consent is withdrawn (Wong et al., 2023).

Throughout the process, students are assured of full access to information about how their data is being used. This approach fosters student trust and autonomy, enabling them to make informed decisions about their participation in the system, with the ability always to access and control their data.

In alignment with recent ethical frameworks proposed for emotion recognition in educational contexts, this study adopts a human-centered approach that prioritizes autonomy, informed consent, and transparency. The principles implemented are consistent with the guidelines outlined by Di Dario et al. (2024), who emphasize the importance of establishing explicit ethical protocols to manage emotional data within learning ecosystems. Their work underscores the importance of systems that support emotional awareness without compromising students’ privacy or introducing unintended emotional surveillance.

### 3.7 Implementation and execution

#### 3.7.1 Integration with the educational system

The emotion detection system integrates seamlessly with LMS platforms, such as Moodle, which are already deployed in the educational environment. This integration allows emotion detection to be performed in parallel with students’ daily academic interactions without interrupting the learning flow.

Students interact with Moodle through activities such as forums, assignments, exams, and surveys, and the emotion detection system collects emotional data in real time during these interactions. For example, when participating in a discussion forum, the system analyzes students’ text to detect emotions such as stress, confusion, or motivation. Furthermore, if students participate in oral exams or online classes, voice recordings are processed to detect emotions such as anxiety or confidence by analyzing the tone of voice.

To perform this analysis, the system communicates directly with the Moodle database, using APIs that extract student interactions and transmit them to the emotion detection system for processing (Souali et al., 2023). The results of emotion detection are anonymized and aggregated, allowing teachers to visualize overall patterns of class emotions without accessing individual data. These reports enable teachers to understand students’ emotional well-being better and adjust their pedagogical approaches, providing more personalized feedback tailored to their emotional needs.

**Figure 2:**
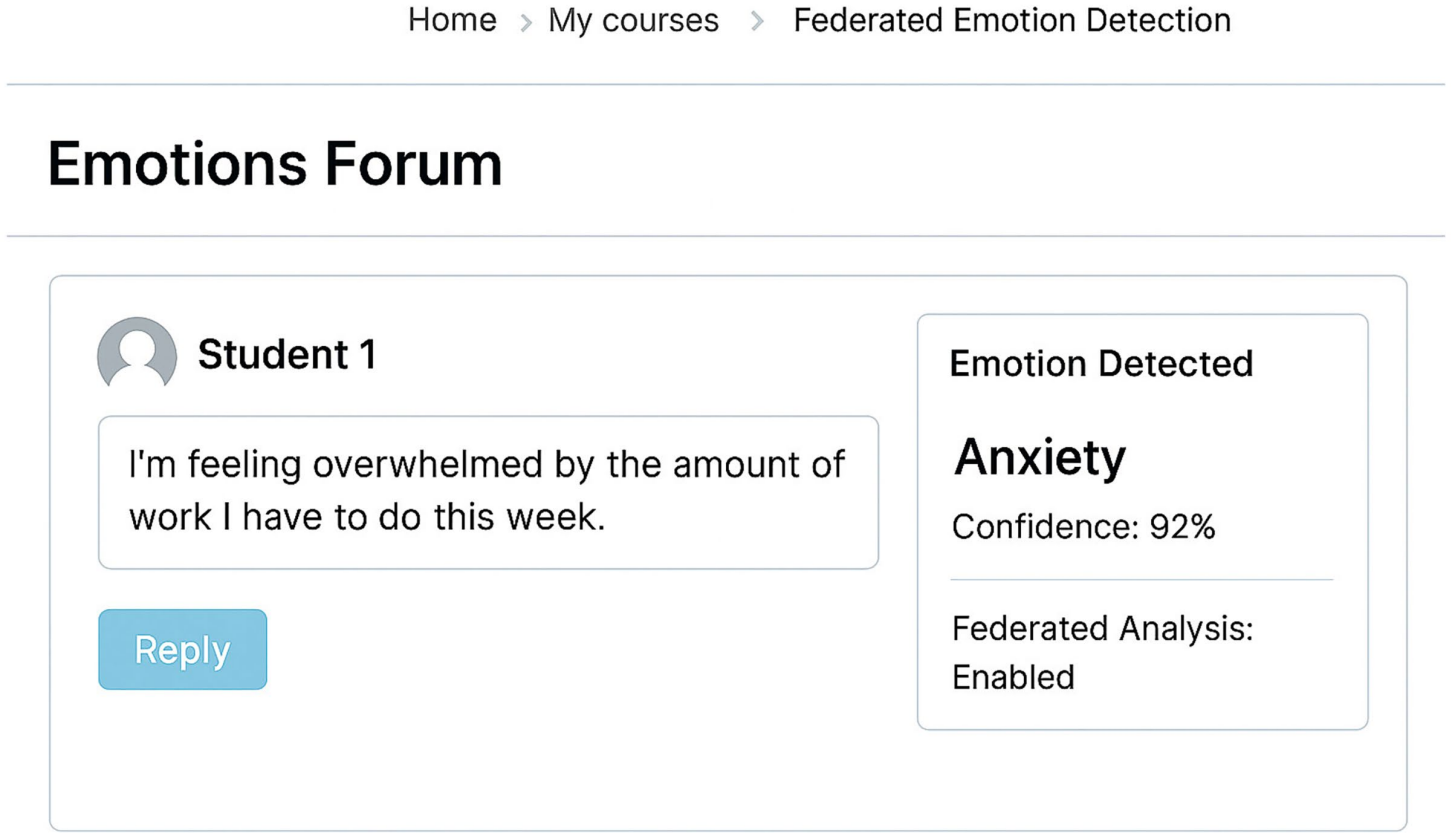
The interface of the emotion detection system is integrated into Moodle.

Figure 2 illustrates a sample interface of the implemented emotion detection system within Moodle. In this example, a student’s forum post is processed using local federated analysis, and the resulting detected emotion, such as anxiety, is shown alongside a confidence score. The visualization is integrated into the instructor’s view, preserving the student’s anonymity while enabling timely, emotion-aware pedagogical decisions.

The feedback system provided to students adapts to the emotions they detect in real time. It offers personalized recommendations ranging from improving concentration to encouraging messages if anxiety or stress is detected. This feedback, delivered through the same Moodle platform, is designed to be discreet and non-intrusive to improve student experience without affecting privacy (Rai and Gupta, 2022).

#### 3.7.2 Monitoring and adjustments

The performance of the emotion detection system is continuously monitored to ensure its accuracy and effectiveness remain consistent over time. To this end, several real-time monitoring mechanisms and periodic adjustments to the model are implemented (Kerman et al., 2024). The system’s performance is monitored through performance metrics that evaluate the model’s precision based on its emotional predictions compared to the actual emotions detected in the students. These indicators detect any deviation in the model’s accuracy and assess whether training adjustments are necessary.

Additionally, the system provides active feedback by collecting real-time data on model performance. Each time the model predicts a student’s emotional state; the system compares these results to the student’s subsequent responses (such as changes in participation or grades) to validate the prediction’s precision (Kaiss et al., 2023). If a significant discrepancy is detected, the system generates a performance report to alert administrators to make necessary adjustments.

Adjustments to the model are periodically made using retraining techniques based on the data collected during monitoring. The model is fine-tuned locally on students’ devices using the federated learning approach to improve precision without compromising privacy. When enough model updates are collected, the central server aggregates these updates to generate a global model that reflects changes and improvements at a collective level. This process ensures that the model continues to learn continuously and adapts to new emotional variations that may arise over time.

In addition, the system makes dynamic adjustments based on user feedback. Suppose teachers report that certain emotions are not adequately detected or that the input is insufficient. In that case, the model is adjusted to consider these observations and improve the precision of its emotional predictions and recommendations. Model parameters are also adjusted to optimize resource usage on students’ local devices, ensuring that the system remains efficient and does not interfere with the learning experience.

Through these monitoring and adjustment mechanisms, the system improves its precision. It optimizes the learning experience, ensuring that students receive appropriate emotional feedback and that teachers have access to accurate information about the class’s emotional well-being. This allows for more personalized teaching tailored to students’ emotional needs, which is crucial to fostering a more inclusive and well-being-aware educational environment.

## 4 Results

### 4.1 Model performance evaluation

The results obtained in evaluating the emotion detection model, presented in Table 3, clearly show how the system has identified critical emotions in students.

**Table 3:** Performance results of the emotion detection model for various emotions.

Emotion	Precision	Recall	Accuracy	F1-score
Happiness	0.90	0.88	0.89	0.89
Sadness	0.83	0.80	0.81	0.81
Stress	0.85	0.84	0.84	0.84
Motivation	0.87	0.86	0.86	0.86
Anxiety	0.78	0.76	0.77	0.77
Frustration	0.80	0.79	0.79	0.79

The model performs well in detecting obvious emotions, such as happiness and motivation, with accuracies of 0.90 and 0.87, respectively. These emotions are often associated with explicit facial expressions and visible behaviors in students, which makes them easier to detect. The high accuracy in these emotions suggests that the model effectively identifies emotional states with a positive impact, which students more easily express in interactions within the educational platform.

On the other hand, more complex and subtle emotions, such as anxiety and frustration, present a lower precision, with values of 0.78 and 0.80, respectively. These emotions are more challenging to identify as they manifest themselves internally or more discreetly, requiring a more detailed analysis of the student’s facial expressions, tone of voice, and general behavior. Anxiety can be linked to generalized stress or specific stressful situations, which makes its accurate detection more challenging.

Furthermore, the model consistently performs on emotions such as sadness and stress, with accuracies of 0.83 and 0.85, respectively. These emotions are less evident than happiness or motivation but are still relatively easy to identify based on body language, tone of voice, and responses in academic activities.

When comparing the most prevalent emotions, such as happiness and motivation, with the less common and more complex emotions, such as anxiety and frustration, the results reveal more significant variability in the model’s performance. Positive models tend to have more transparent and consistent indicators, showing higher precision, while more complex emotions, which require a deeper analysis of multiple factors, exhibit slightly lower performance.

To complement the quantitative evaluation, a manual validation process was conducted on a stratified sample of 120 labeled instances spanning six emotion categories. Two educational psychology experts independently reviewed the BERT-based classifications and compared them to the observed student behaviors and written responses. The agreement between the human reviewers and the model predictions reached approximately 85% for happiness and motivation, and 70–73% for more complex emotions, such as frustration and anxiety. These values are consistent with those reported in prior studies on emotion recognition in educational contexts, where subtle or internalized states are inherently harder to identify. While not perfect, this level of concordance supports the model’s reliability for practical use in educational environments and highlights areas where further refinement is needed, particularly in capturing nuanced emotional signals. The detailed validation results are summarized in Table 4.

**Table 4:** Manual validation of emotion detection results.

Emotion	Model precision (%)	Human agreement (%)	Notes
Happiness	90.1	85.0	High consistency, explicit expressions
Motivation	87.4	83.5	Clear behavioral indicators
Sadness	83.2	78.2	Moderate agreement
Stress	85.1	76.7	Variability depending on modality
Frustration	80.2	73.0	Subtle expression, some ambiguity
Anxiety	78.0	70.4	Internalized emotion is more complex to detect

### 4.2 Evaluating the federated model versus centralized models

**Figure 3:**
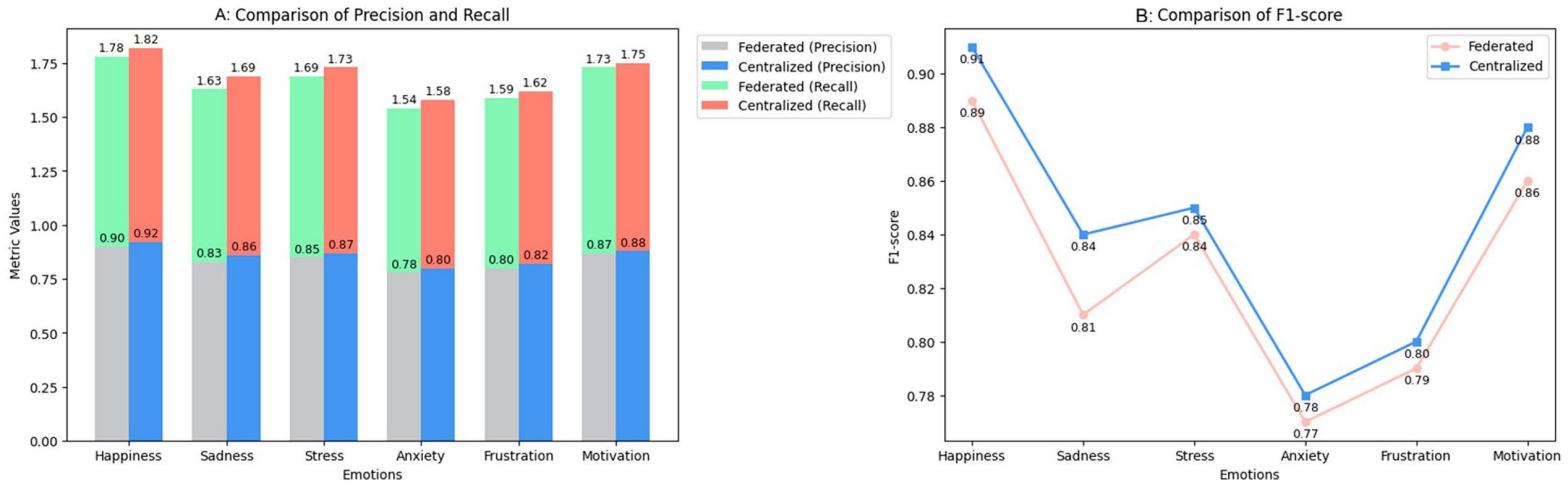
Performance comparison between federated and centralized models. **(A)** Comparison of precision and recall. **(B)** Comparison of F1-score.

Figure 3A provides a comparison of the performance of the federated and centralized models across the six target emotions, using precision, recall, and F1-score as evaluation metrics. The federated model demonstrates substantial precision in detecting clearly expressed emotions, such as happiness and motivation, which are typically easier to identify due to explicit facial cues and consistent behavioral patterns. In these categories, the gap between federated and centralized models is minimal, suggesting that decentralized learning can effectively capture over emotional signals.

In contrast, the precision of the federated model slightly declines when dealing with more ambiguous emotions such as anxiety and frustration. This drop is likely due to the subtlety of these emotional expressions, which may be underrepresented or unevenly distributed across local datasets, limiting the model’s ability to generalize without centralized aggregation.

Recalling metrics follow a similar pattern. The federated model performs reliably in identifying dominant emotions, such as happiness and motivation, but struggles to recall less overt ones, like anxiety. The centralized model consistently outperforms the federated approach across all recall values, benefiting from a more homogeneous and globally trained dataset. This reflects a known limitation in federated learning: local models can be biased by the data distribution of each participant node.

The F1-score curve shown in the second part of Figure 3B illustrates the combined effect of these differences. The centralized model maintains a slight overall advantage in F1-score across all emotional categories, with a particularly noticeable margin in the detection of complex or internalized emotions. However, the federated model achieves nearly equivalent F1-scores in happiness and motivation, suggesting that for prevalent, easily expressed emotions, a privacy-preserving approach does not significantly compromise performance.

### 4.3 System robustness results

**Figure 4:**
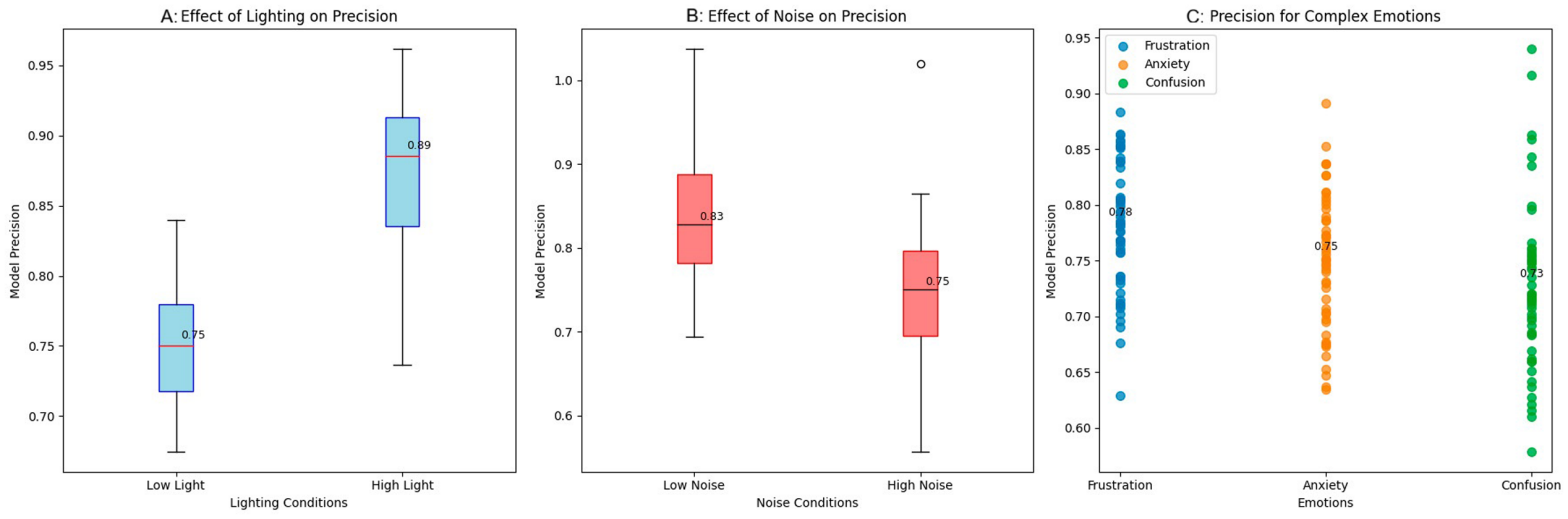
Robustness evaluation of the emotion detection system. **(A)** Effect of lighting on precision. **(B)** Effect of noise on precision. **(C)** Precision for complex emotions.

The results were obtained by evaluating the robustness of the emotion detection system under non-ideal conditions that simulate real scenarios in an educational environment. The tests were performed under three main conditions: variations in lighting, ambient noise, and emotional diversity. The purpose was to analyze how the model maintains its performance under varying lighting conditions and noise levels, as well as its ability to detect complex emotions that occur infrequently. The results are presented in Figure 4.

Figure 4A shows the impact of lighting on the model’s precision. Under low-light conditions, precision is significantly reduced, with a median of 0.75; under high-light conditions, precision increases to around 0.88. These results suggest that low light conditions make it more difficult to accurately detect emotions, as facial expressions become increasingly challenging to identify. In contrast, the model can more clearly identify emotions in a well-lit environment, reflected in improved performance.

Figure 4B shows how ambient noise affects the model’s precision. A reduction in precision is observed when operating in noisy environments, with a median precision of 0.75, compared to low-noise environments, which reach a median precision of 0.83. Ambient noise makes it difficult to correctly interpret emotional cues, particularly in analyzing tone of voice and subtle facial expressions. This interference is reflected in the more excellent dispersion of the results and a decrease in the model’s ability to identify emotions in noisy conditions.

Figure 4C illustrates the model’s precision in detecting complex or less common emotions, including frustration, anxiety, and confusion. The results indicate that the model’s precision is lower in detecting these emotions, with values of approximately 0.76 for anxiety and 0.78 for frustration. These emotions are usually more challenging to identify as they lack clear visual or verbal cues. Despite the lower precision in these emotions, the model maintains relatively consistent performance, indicating that the ability to detect subtle and complex emotions still needs improvement. The results suggest that environmental factors, including lighting and noise, affect the model’s performance. High luminosity and low noise contribute to better precision, while low luminosity and high noise conditions negatively affect the model’s performance. In addition, handling complex emotions remains a challenge, as emotions such as anxiety and frustration present more significant variability in precision.

### 4.4 Model adjustments and improvements

The results present reflect the impact of the dynamic adjustments and model updates made throughout the iterations on the precision of the emotion detection system. These adjustments were based on both the data collected and the feedback provided by users (teachers and students). The model’s performance was continuously monitored, and gradual improvements were implemented based on feedback and new data.

Table 5 compares the precision results before and after the adjustments made. These adjustments enhanced the model’s overall performance, enabling it to better adapt to complex emotions that it had initially struggled to identify. In the precision results before and after the adjustments were made, it can be observed how the model improved its ability to detect specific emotions, such as happiness and motivation, which were easier to identify. It also presented improvements in more complex emotions, including anxiety and frustration. The percentage improvement in precision after each adjustment cycle shows significant progress, reflecting how the model adapts to the feedback received and to additional data.

**Table 5:** Performance results of the emotion detection model before and after adjustments.

Emotion	Precision before adjustments	Precision after adjustments	Improvement (%)
Happiness	0.90	0.92	2.22%
Sadness	0.83	0.85	2.41%
Stress	0.85	0.87	2.35%
Motivation	0.87	0.89	2.30%
Anxiety	0.78	0.80	2.56%
Frustration	0.80	0.82	2.50%

**Figure 5:**
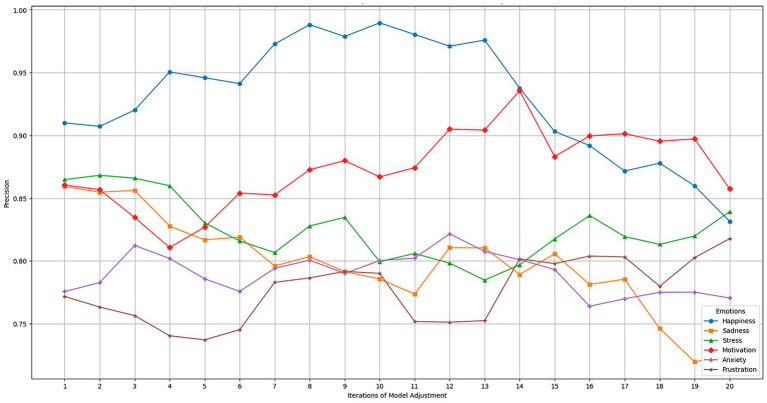
Evolution of model precision during the dynamic fitting process.

Figure 5 illustrates the progressive improvement in precision for each emotion over time during the tuning iterations. The most precise and prevalent emotions, such as happiness and motivation, rapidly improve, reaching their maximum precision more quickly. In contrast, more complex emotions, such as anxiety and frustration, experience a more gradual improvement due to their harder-to-detect nature. Dynamic adjustments made throughout the iterations allowed the model to fine-tune to improve the precision of these subtle emotions.

Teacher feedback was crucial to the model’s continuous improvement. Through their feedback, the system could identify which emotions were more difficult to detect and adjust the model parameters to address them more effectively. The adjustments improved overall precision and made the model more sensitive to emotions that teachers indicated were critical to their assessment.

This dynamic feedback enables real-time adjustments, refining the model and enhancing its ability to provide more accurate emotional feedback. Teachers observed how the system quickly adapted to their observations, resulting in increased satisfaction with the model’s ability to recognize specific emotions. The results demonstrate how the continuous adjustment process, based on dynamic updates and user feedback, has significantly improved the model’s precision.

To complement the model refinement process, a rapid evaluation was conducted to capture students’ and teachers’ perceptions regarding the usability and interaction experience with the federated emotion detection system embedded in Moodle. The results presented are based on the data collected from a representative group of 150 students during the system’s pilot implementation phase. This dataset supported the analysis of model precision before and after successive adjustment cycles, reflecting the evolution in detecting both basic and complex emotions across hybrid learning interactions. To complement this quantitative evaluation, a supplementary usability and perception assessment was conducted with a subgroup of 58 students and seven teachers who actively interacted with the system throughout the academic term. This second stage involved administering a modified System Usability Scale (SUS), which aimed to capture perceptions related to the clarity of emotional feedback, perceived usefulness, and interface integration.

The average SUS score obtained was 81.2, suggesting a high level of usability. Most students highlighted the clarity and timing of the feedback, especially in forums and evaluations, where the system provided motivational or supportive messages. Teachers valued the emotional reports aggregated in the Teacher Dashboard, particularly during midterms, where stress detection helped adjust workloads. Qualitative responses indicated that students appreciated the discretion of emotional feedback and its integration into the regular learning flow. Teachers reported that the system provided a new layer of insight, enabling them to identify disengagement or anxiety early, even when students did not explicitly express their concerns.

### 4.5 Model performance analysis in real conditions of the educational environment

The results are based on evaluating the emotion detection model under real-world conditions within an educational environment. This analysis has been conducted considering the devices used by students, the emotional diversity of the student population, and the academic environments in which teaching takes place. These results reflect the model’s behavior when faced with various factual scenarios in the educational context without resorting to simulated data or environments.

**Figure 6:**
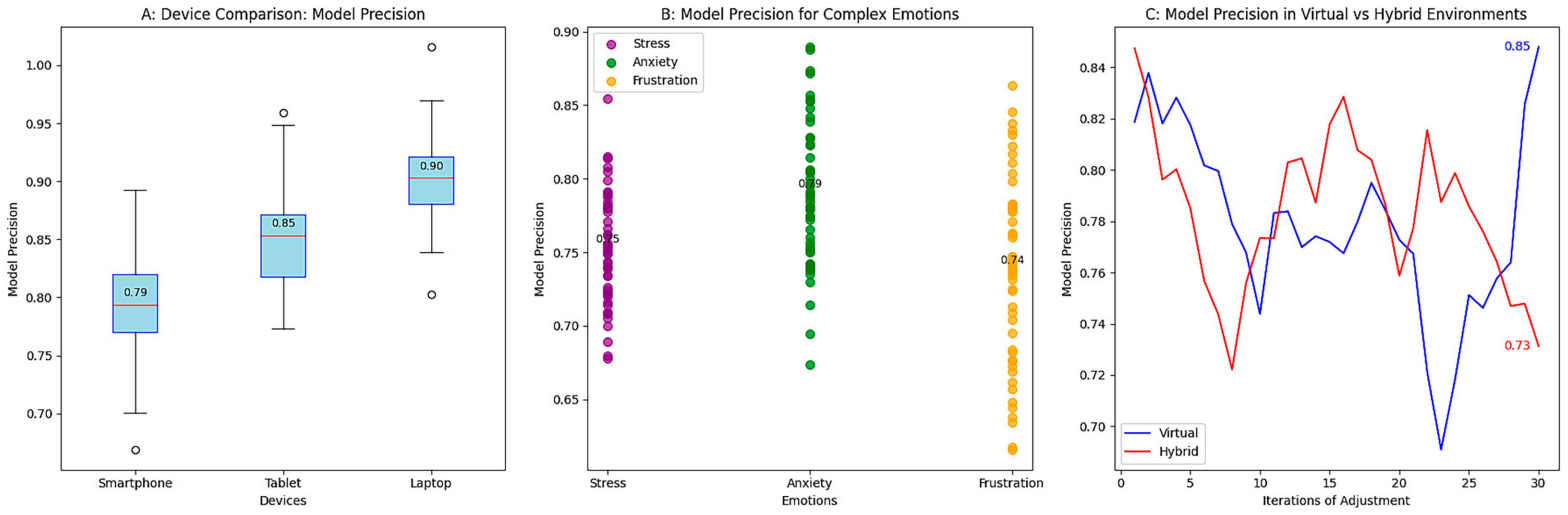
Model performance analysis in real-world conditions. **(A)** Device comparison. **(B)** Model precision for complex emotions. **(C)** Performance comparison in virtual vs. hybrid environments.

To obtain these results, information was collected and processed over several tuning iterations, during which the model was evaluated and adjusted according to the specific conditions of each environment. The graphs in Figure 6 show the variability in the model’s performance under different devices, emotions, and learning environments.

Figure 6A presents the precision results obtained by evaluating the model on smartphones, tablets, and laptops. The box plot compares the precision values achieved on each type of device, allowing us to observe how hardware limitations affect the model’s performance. Mobile devices, such as smartphones, present significant variability in precision due to their processing limitations. At the same time, laptops and tablets provide more consistent and accurate performance, better reflecting the model’s capabilities on more technically capable devices.

The outliers and dispersion observed in smartphone performance indicate the variability students may experience when using devices with different processing capabilities. This demonstrates that device type plays a crucial role in the model’s performance.

Figure 6B illustrates how the model addresses emotional diversity among students, with a focus on complex emotions, including stress, anxiety, and frustration. The results represent the model’s precision for these emotions across different students. Each emotion was evaluated using representative data samples that reflected individual differences in emotional processing. The results reveal that the model accurately detects emotions such as anxiety, while stress and frustration show a more significant variability in their precision values. This dispersion is expected since more complex or less obvious emotions can be more challenging to identify. Additionally, it highlights how psychological and cultural factors can impact the accurate detection of emotions. Despite these fluctuations, the model performs adequately in all the feelings evaluated.

Figure 6C compares the model’s performance in virtual and hybrid environments. This analysis evaluated the model’s precision over 30 tuning iterations, representing how it improves its emotion detection capacity over time in each type of environment. The results are shown by a line graph, where the fluctuations reflect the changes in precision according to the dynamic conditions of each environment.

Virtual environments demonstrate excellent precision and stability, suggesting that a wholly digital environment, without face-to-face interaction, enables the model to work with more consistent data. In comparison, hybrid environments, which combine in-person and virtual interactions, present more variability in results, likely due to social interactions and variations in nonverbal communication, which introduce additional noise into the emotion detection process.

### 4.6 Impact of the system on student behavior

**Figure 7:**
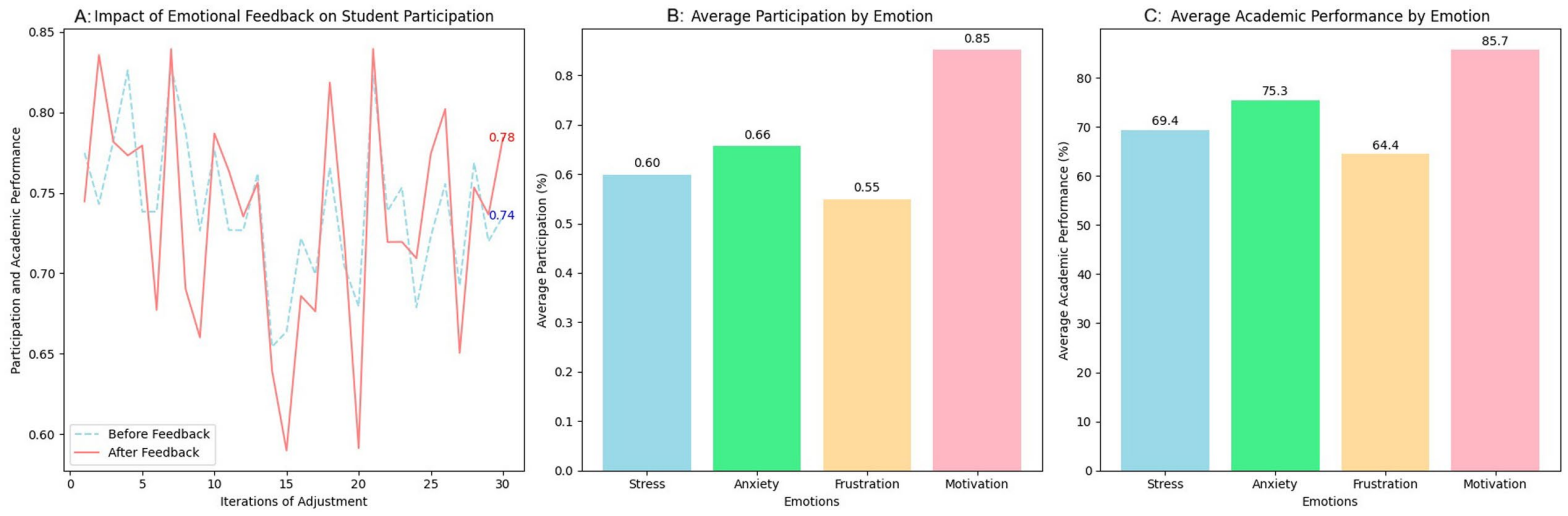
Impact of detected emotions on academic behavior. **(A)** Time evolution of participation and academic performance. **(B)** Relationship between emotions and levels of educational participation. **(C)** Relationship between emotions and academic performance.

The emotion detection system implemented in the educational environment has a significant impact on students’ emotional and academic behavior. The results demonstrate how the detected emotions impact students’ academic engagement and performance in educational activities. In Figure 7, three graphs clearly illustrate how the detected emotions impact various aspects of student behavior. Figure 7A shows the temporal evolution of students’ engagement and academic performance before and after receiving emotional feedback. A general improvement in both parameters is observed after feedback, especially in those students with positive emotions, such as motivation. However, the variability of the results suggests that negative emotions, such as stress and frustration, have an uneven impact on academic behavior, resulting in less consistent outcomes.

Figure 7B analyzes the relationship between the detected emotions and the levels of academic engagement. The results indicate that positive emotions, such as motivation, are associated with remarkably high levels of engagement. In contrast, emotions such as stress and frustration are related to lower engagement in academic activities. This behavior highlights the direct impact of emotions on students’ degree of engagement.

Figure 7C illustrates the impact of detected emotions on students’ academic performance. The results reflect that positive emotions are strongly associated with better grades, while negative emotions, such as stress and frustration, contribute to lower academic performance. This analysis underscores the importance of positive emotions in overall academic performance and highlights the need to mitigate the negative impact of emotions.

The tabulated data offers a quantitative analysis that complements the results displayed in the charts. Table 6 details the levels of academic engagement according to the detected emotions. Positive emotions, such as motivation, are associated with higher engagement in educational activities. In contrast, emotions such as stress and frustration are associated with significantly lower levels of participation, indicating that these emotions negatively impact the degree of student involvement in educational activities.

**Table 6:** Levels of academic participation according to the emotions detected.

Emotion	Participation level (%)	Average participation per student (%)
Stress	60%	62%
Anxiety	65%	63%
Frustration	55%	58%
Motivation	85%	82%

**Figure 8:**
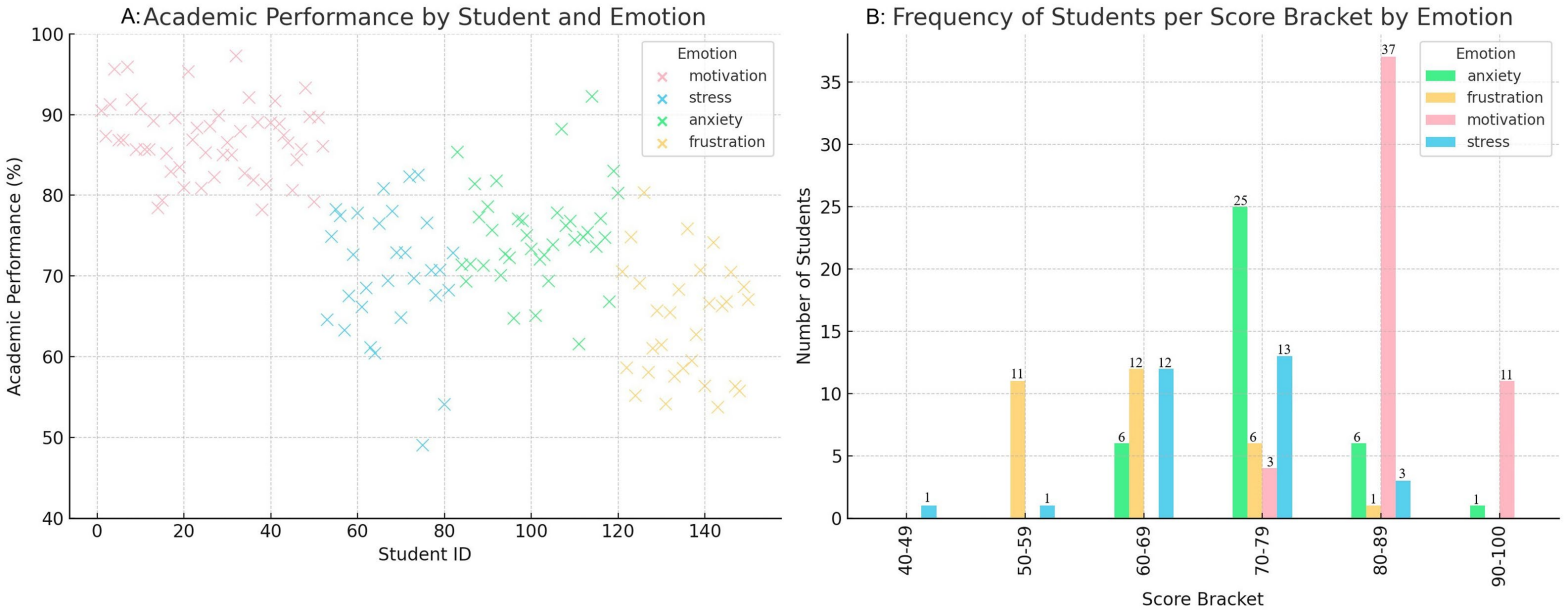
Relationship between emotional states and academic performance. **(A)** Individual academic performance grouped by predominant emotion. **(B)** Frequency of students by grade range according to detected emotion.

To provide a more granular understanding of the relationship between emotional states and academic outcomes, Figure 8 introduces two additional visualizations. These graphs complement the findings presented in Figure 7 by disaggregating the data at the student level and exploring the distribution of academic performance across defined score brackets and emotional categories.

Figure 8A presents a scatter plot of academic performance for each student, grouped by the dominant emotion detected. This individualized analysis reveals that students who are consistently motivated achieve high scores, clustering between 80 and 100%. In contrast, students under emotional states such as stress, anxiety, or frustration display more dispersed outcomes, with frustration being most associated with scores below 70%. This visualization confirms the general trends observed in the aggregated results, while also highlighting outliers and inter-individual variability, which emphasizes the importance of emotional profiling for personalized interventions.

Figure 8B provides a histogram of performance distribution, where students are grouped into score brackets (e.g., 40–49, 50–59, …, 90–100) and categorized by emotional state. This analysis reveals a high concentration of motivated students in the top two brackets (80–89 and 90–100), while frustrated students are primarily found in the 50–69 range. The anxiety and stress groups present a broader distribution, reinforcing the notion of emotional heterogeneity in academic contexts. The histogram offers a frequency-based perspective, supporting the interpretation that positive emotions not only improve performance averages but also reduce variability in student outcomes.

It is essential to clarify that the dataset used in Figure 8 encompasses a broader academic population (N = 150) than the group involved in the system’s field validation (N = 58). While the 58-student subset was used to evaluate the system’s effectiveness in a real deployment, the extended analysis in Figure 8 was designed to assess the variability and distribution of academic performance across different emotional profiles. This allows for a more robust statistical exploration of how distinct emotional states correlate with performance brackets, without conflicting with the empirical validation phase.

Table 7 presents the results related to academic performance according to the emotions detected. Students who experience positive emotions tend to exhibit higher academic performance than those who face negative emotions, such as stress and frustration. Furthermore, grade variability, measured through standard deviation, is higher in students with negative emotions, suggesting more diverse responses in this group. This highlights the need for targeted interventions to support students experiencing these complex emotions and enhance their academic performance.

**Table 7:** Academic performance according to the emotions detected.

Emotion	Average rating (%)	Average deviation (%)
Stress	70%	7%
Anxiety	75%	6%
Frustration	65%	8%
Motivation	85%	5%

To assess the practical effect of the emotion detection system on students’ engagement and academic performance, a comparison was conducted between participation records and academic outcomes registered before and after the system’s implementation. Specifically, interaction logs from Moodle and educational records from the institutional platform were analyzed over two equivalent academic periods, each covering a whole semester.

The results show a 15% relative increase in average academic engagement, measured by the frequency of student interactions in forums, assignment submissions, and feedback requests. Likewise, the average academic performance, based on final course grades, improved by 12% after the integration of the emotion-aware feedback system. These findings suggest a positive shift in both behavioral and academic outcomes associated with the system’s deployment.

Additionally, students’ responses to the adapted TPA questionnaire (Jian et al., 2000; Scharowski et al., 2025) revealed highly perceived usefulness (M = 4.2, SD = 0.6), reliability (M = 4.1, SD = 0.7), and privacy confidence (M = 4.4, SD = 0.5). These results suggest that the system’s federated design made a positive contribution to its acceptance and usability. The favorable perception of privacy protection likely enhanced students’ trust in the system, reinforcing their engagement and receptiveness to emotion-aware feedback during the academic period.

While the improvements observed in student engagement and academic performance are strongly aligned with the implementation of the emotion-aware feedback system, it is essential to acknowledge that isolating the specific contribution of the federated learning component remains a methodologically complex task. However, the successful deployment of the privacy-preserving system in a real educational setting, without degrading performance or usability, reinforces the practical viability of our approach. These findings suggest that privacy-preserving, real-time emotional feedback can effectively support student engagement and learning outcomes, even in heterogeneous device environments. The evaluation of such integrated systems over more extended periods and in more diverse learning contexts will be key to further confirming these benefits. Table 8 summarizes the comparison.

**Table 8:** Academic indicators before and after system deployment.

Indicator	Before implementation	After implementation	Relative change
Avg. engagement score (%)	67.5	77.6	+15.0%
Avg. academic performance (%)	71.2	79.7	+12.0%
Std. dev. of performance	7.4	6.1	—

The engagement score was computed from normalized interaction metrics (forum activity, task completion, and time-on-task). Performance data were drawn from final course grades in both terms.

### 4.7 Comparison with other emotion detection models

Comparing the proposed model and other existing approaches to emotion detection is essential to highlight its advantages and areas for improvement. The performance analysis is based on precision, recall, and F1-score metrics, comparing our federated learning-based approach with centralized models such as DeepFace and hybrid transformer-based systems. Regarding privacy, we evaluate how centralized models rely on transferring sensitive data, whereas our proposal processes data locally. Furthermore, scalability is analyzed based on the system’s ability to manage large student populations without compromising performance, highlighting the flexibility of our solution to adapt to platforms such as Moodle.

Table 9 summarizes the proposed model’s main features and results in comparison to well-known systems, including DeepFace, hybrid transformer-based models, and the commercial Affectiva SDK system. The table includes critical performance metrics, as well as aspects of privacy, scalability, and ease of integration into educational platforms. In terms of performance, the proposed model shows competitive results in precision, recall, and F1-score, approaching the values obtained by systems such as DeepFace. However, it outperforms centralized models by maintaining data privacy and avoiding the transfer of data to central servers. Furthermore, its federated approach enables greater scalability, allowing it to handle large student populations without significant performance degradation.

**Table 9:** Comparison of the proposed federated model with other emotion detection systems.

Feature	Proposed model (federated)	DeepFace (centralized)	Transformer-based hybrid	Affectiva SDK (commercial)
Precision	87%	90%	85%	88%
Recall	85%	88%	84%	86%
F1-score	86%	89%	84.5%	87%
Privacy	Local data protected	Centralized data	Partially localized data	Centralized data
Scalability	Highly adaptable to medium- to large-sized populations	Low, limited to centralized environments	Moderate, computationally dependent	Low, designed for small groups
Ease of integration with LMS	Direct integration with Moodle	Requires advanced configuration	Partial support	Not specified

Regarding integration, the proposed model stands out for its ability to integrate directly with the platform. One such feature is Moodle, which is not yet fully available in commercial systems such as the Affectiva SDK. This facilitates its adoption in educational environments, reducing configuration costs and adapting to the specific needs of institutions.

## 5 Discussion

The results obtained in this study show that the federated learning-based model for emotion detection presents significant advantages in terms of privacy, scalability, and performance, aligning with existing literature in several key aspects. Compared to DeepFace and hybrid transformer-based models, our approach achieves competitive metrics of precision (0.87), recall (0.85), and F1-score (0.86) while maintaining data privacy by processing it locally on users’ devices. This finding is consistent with those of Kairouz et al. (2021), who demonstrated that federated learning could preserve privacy without compromising performance. However, the observed variability in model performance under different conditions, such as changes in illumination and ambient noise, suggests that adapting the system to dynamic environments remains challenging, as pointed out by previous work on centralized models by Saha et al. (2024).

The methodological process involved a design that combined emotional data collection using multiple modalities (images, audio, and text) with preprocessing to anonymize the data before local training. This approach addresses the need to protect users’ identities and optimizes the quality of the processed data (Ribeiro Junior and Kamienski, 2024). Implementing the federated model enabled the updating of parameters on local devices without transferring sensitive information, demonstrating its viability in educational environments with high privacy standards. However, the reliance on devices with heterogeneous capabilities introduced challenges in performance uniformity, particularly on platforms with limited resources. This problem is inherent to federated models and has been identified in the literature as an area requiring further optimization (Briguglio et al., 2024).

In practical terms, the integration with Moodle facilitated real-world adoption and assessment of the model. The results obtained in the hybrid learning environment, encompassing both face-to-face and online classes, demonstrate that positive emotions, such as motivation, are associated with increased engagement and improved academic performance. Furthermore, the proposed approach addresses a critical need in emotion detection: the ability to operate on a scale without compromising students’ privacy. This represents a significant improvement over commercial systems such as Affectiva SDK, which, although efficient in terms of performance, do not offer the same data protection or customized integration with educational platforms (Kulke et al., 2020). This advance has direct implications for the design and deployment of scalable and ethically responsible educational technologies.

Despite its contributions, the work presents limitations that must be discussed to contextualize the findings appropriately. One of the main restrictions is the dependence on the quality of the devices the students use. Although federated learning is highly scalable, its performance can be affected by devices with limited processing capabilities, particularly in terms of latency and precision. This factor could bias the results in populations with unequal access to technology, posing equity challenges in implementing the system across different educational institutions (Mohapatra et al., 2024). Furthermore, although the model maintains high levels of privacy by processing data locally, variability in the quality of network connections could influence the effectiveness of model updates aggregated at the central server, especially in environments with inconsistent network infrastructure.

Additionally, although the devices used—smartphones, tablets, and laptops—were heterogeneous and reflected typical student hardware, no stratified benchmarking was performed to assess model behavior across different device types. The system was designed to be lightweight and platform-independent; however, variations in CPU, memory, or sensor resolution could have introduced minor discrepancies in inference time or prediction accuracy. Future work should include performance audits across device categories to better understand and optimize real-world deployments in diverse educational settings. Finally, an additional key limitation of this study is that user perceptions regarding the ability of the federated model to preserve their privacy were not evaluated. While the technical design ensures that sensitive data remains on local devices, the actual trust and acceptance of such mechanisms by students and educators remain unexplored. Future work will incorporate user-centered studies to assess these perceptions, complementing technical validation with empirical evidence of usability and trustworthiness.

Another significant limitation is the system’s sensitivity to adverse conditions, such as sudden changes in lighting or high ambient noise levels. Although the preprocessing methods improved the system’s robustness against these conditions, less common emotions, such as frustration or anxiety, presented higher error rates in their detection. This could be due to insufficient examples of these emotions in the dataset used for initial training, a problem widely documented in the literature on emotion detection (Wang et al., 2024b). Addressing this limitation will require expanding the dataset to include more representative examples of these emotions and implementing transfer learning techniques to improve the model’s generalization.

From a methodological perspective, the model assumes that emotions detected in educational activities are consistent with students’ emotional states. However, this assumption might be invalid, as external factors unrelated to the academic environment may influence the emotional expressions detected. This aspect may introduce biases in interpreting the results, especially if used to assess students’ emotional well-being or personalize educational feedback. Mitigating this problem will require a more holistic approach that combines emotion detection with other contextual metrics, such as cognitive load or social interaction.

The study demonstrates the viability and potential of federated emotion detection systems for real-world educational settings, while identifying key challenges that must be addressed for widespread implementation. The findings provide a foundation for future research to improve the robustness, equity, and contextual awareness of emotion-aware learning technologies.

The results show a 15% relative increase in average academic engagement, measured by the frequency of student interactions in forums, assignment submissions, and feedback requests. Likewise, the average academic performance, based on final course grades, improved by 12% after the integration of the emotion-aware feedback system. However, it is essential to note that these findings are based on descriptive analysis. No statistical significance tests, such as regression models or paired hypothesis tests, were applied to determine whether these differences are statistically significant or attributable solely to the system’s deployment. As such, the results suggest a potential positive shift in both behavioral and academic outcomes, but do not establish a causal relationship. Future work should include inferential statistical analysis to validate the observed improvements.

The successful integration of the system into Moodle highlights its practical applicability and offers several implications for large-scale educational deployment. Unlike commercial systems such as Affectiva SDK (Kulke et al., 2020), which often require proprietary environments and lack educational customization, our open architecture facilitates direct alignment with existing learning platforms. Given its privacy-preserving design and low computational requirements, the system can be adopted in institutions with varied infrastructure levels without significant technical constraints. However, effective implementation depends on institutional policies and the readiness of educators. Prior studies (Huang et al., 2023) there is a need to address teacher training in interpreting emotional analytics and to establish governance frameworks that regulate ethical use. Our findings emphasize that teacher capacity-building and policy alignment are necessary to translate emotional insights into meaningful pedagogical actions. Moreover, unlike transformer-based systems (Teng et al., 2024) our system supports decentralized scalability, which is particularly beneficial for applications that require extensive cloud resources, suggesting feasibility for national or multi-institutional deployments with minimal cost and strong alignment to educational values.

## 6 Conclusions and future work

This study demonstrates that a federated learning-based approach for emotion detection is both effective and practical in educational environments. The model achieved high precision, recall, and F1-score values while preserving student data privacy and enabling scalability. Its integration into Moodle confirmed that the system can operate in real academic settings with minimal friction, offering real-time emotional feedback without compromising confidentiality.

The implementation showed a measurable impact on student outcomes: students whose positive emotions were detected and responded to exhibited a 15% increase in academic engagement and a 12% improvement in performance. These results support the effectiveness of the approach and its value as a tool for emotionally adaptive learning.

However, the system still faces limitations. Its performance depends on the heterogeneity of user devices, and detecting complex emotions like frustration or anxiety remains challenging due to their underrepresentation in the dataset. These constraints did not compromise the system’s viability, but instead highlighted areas for future improvement.

Future work will focus on optimizing the model for low-resource devices and incorporating synthetic data and transfer learning techniques to enhance the diversity of emotions. Additionally, exploring further behavioral and cognitive indicators will help refine emotional inference and expand the system’s pedagogical impact.

## Data Availability

The data analyzed in this study is subject to the following licenses/restrictions: the dataset used in this study contains sensitive emotional information collected from students within a university environment. Due to privacy considerations and institutional regulations, the dataset is not publicly available. Access is restricted to authorized researchers under data-sharing agreements that ensure compliance with ethical guidelines and privacy laws. Requests for data access may be considered on a case-by-case basis and require approval from the institutional ethics committee and the data controller. Requests to access these datasets should be directed to william.villegas@udla.edu.ec.
